# Effect of Mediterranean Diet in Diabetes Control and Cardiovascular Risk Modification: A Systematic Review

**DOI:** 10.3389/fpubh.2015.00069

**Published:** 2015-04-28

**Authors:** Dana Sleiman, Marwa R. Al-Badri, Sami T. Azar

**Affiliations:** ^1^Department of Internal Medicine, Division of Endocrinology, American University of Beirut-Medical Center, Beirut, Lebanon

**Keywords:** Mediterranean diet, dietary pattern, type II diabetes, glycemic control, HbA1c, insulin resistance, cardiovascular risk

## Abstract

**Background:**

Over the past few years, there has been a worldwide significant increase in the incidence of type II diabetes mellitus with both increase in morbidity and mortality. Controlling diabetes through life style modifications, including diet and exercise has always been the cornerstone in diabetes management. Increasing evidence suggests that the Mediterranean diet could be of benefit in diseases associated with chronic inflammation, including metabolic syndrome, diabetes, obesity as well as atherosclerosis, cancer, pulmonary diseases, and cognition disorders As a matter of fact, a number of studies addressed the relationship between Mediterranean diet and diabetes control. The result of these studies was conflicting. Some were able to elicit a protective role, while others showed no added benefit. As a result; we decided to conduct a systematic review to have a better understanding of the relationship between adherence to Mediterranean diet and diabetes control.

**Methods:**

A systematic review was conducted on the effect of Mediterranean diet in diabetes control and cardiovascular risk modification as well as the possible mechanism through which this diet might exhibit its beneficial role. We did a comprehensive search of multiple electronic databases such as Medline, Google Scholars, PubMed, and the Cochrane central register data until May 2014. We included cross-sectional, prospective, and controlled clinical trials that looked at the associations between Mediterranean diet and indices of diabetes control such HbA1c, fasting glucose, and homeostasis model assessment, in addition to cardiovascular and peripheral vascular outcomes.

**Outcome/conclusion:**

Most of the studies showed favorable effects of Mediterranean diet on glycemic control and CVD, although a certain degree of controversy remains regarding some issues, such as obesity. Important methodological differences and limitations in the studies make it difficult to compare results, thus further longer term studies are needed to evaluate the long-term efficacy of the Mediterranean diet along with the possibility of explaining its mechanism.

## Introduction

According to the World Health Organization (WHO) reports, chronic illnesses contribute to 60% of death worldwide (1). Being a major chronic disease; type II diabetes (T2DM) seems to be one of the leading causes of mortality and of public health burden (2). The increasing incidence of diabetes worldwide has been highly linked to the westernized dietary patterns, physical inactivity, and increasing rates of obesity and metabolic syndrome.

Over the past few years, there has been a great effort to study the relationship between dietary patterns and human health. Interestingly, adherence to a healthy life style was strongly associated with reduction in the risk of chronic illnesses (83% reduction in coronary artery disease and 91% reduction in diabetes mellitus in females) (3). Moreover, PREDIMED – Reus study nutrition intervention trial showed that adherence to Mediterranean diet was associated with 52% reduction of diabetes mellitus incidence (4).

The American Diabetes Association (ADA) does not recommend a specific diet over another for the diabetic patients. However, it clearly states that the total amount of fat should represent 25–35%, and saturated fat of <7% of the total calories, with monounsaturated fat as the major source of fat intake. It also points out the importance of adequate amounts of fruits, vegetables, and whole wheat cereals.

On the other hand, the ADA lists three different types of diets (either low-carbohydrate, or low-fat calorie-restricted or Mediterranean diet) as a mean for weight loss for individuals who have or are at risk of having diabetes (5). Because of the abovementioned heterogeneous data and controversial evidence regarding the best dietary pattern in diabetic patients, there was a need to conduct a systematic review to highlight the effect of adherence to a Mediterranean diet on diabetes control and cardiovascular risks.

The term Mediterranean diet was first coined by Ancel Keys in the 1960s. Keys observed the dietary habits adopted by the population residing near the Mediterranean Sea and noticed a reduced incidence of chronic illnesses and higher life expectancy when compared to other regions of the world (6). Defining the term Mediterranean diet is a great challenge given the broad geographical distribution of the Mediterranean countries and the ethnic, cultural, religious, and economic variations among them. However despite this, there is a dietary pattern characteristic of the Mediterranean diet. This includes a diet rich in fruits, vegetables, bread, cereals, olive oil as the major source of fat, low to moderate amounts of fish and poultry and alcohol, and little red meat.

Assessing adherence to Mediterranean diet is not a simple task. For this reason, many indexes were developed to describe the degree of adherence to the Mediterranean diet. Trichopoulo et al. designed a score for assessing the diet adherence (7). In his scoring system, Trichopoulo granted individuals points depending on their daily intake of the separate components of the Mediterranean diet. A score of 9 reflected the highest degree of adherence. Panagiotakos et al. developed another score, which assesses the monthly intake of Mediterranean food rather than the daily one, with a score range of 0–55 (8).

## Methods

In this study, we conduct a systematic review to assess the relationship between the Mediterranean diet and diabetes control. We have searched PubMed, Medline, Google scholars. and the Cochrane central register databases using exploded mesh terms “Mediterranean,” “diet,” “dietary pattern,” “type two diabetes,” “glycemic control,” “HBA1c,” “insulin resistance,” “insulin sensitivity,” “homeostasis model assessment (HOMA),” “Cardiovascular disease,” ”Coronary artery disease “ and “peripheral arterial disease,” “mechanism of action,” and “antioxidant”(Appendix). During our search, *there were no language restrictions*. We also used references from the articles and reviews we extracted to complete the data bank. We identified cross-sectional, prospective, and controlled clinical trials that looked at the associations between Mediterranean diet and indices of diabetes control such as HbA1c, fasting glucose, and HOMA, in addition to cardiovascular and peripheral vascular outcomes. Studies with participants number <15 or follow up period <3 months were excluded. We also excluded studies that assessed adherence to Mediterranean diet subjectively without the use of a score, those with case control design, and those that analyzed adherence to a non-specific dietary pattern rather than a Mediterranean diet.

Our initial search yielded 72 articles, of which 25 were excluded on the basis of abstract.

**Figure 1:**
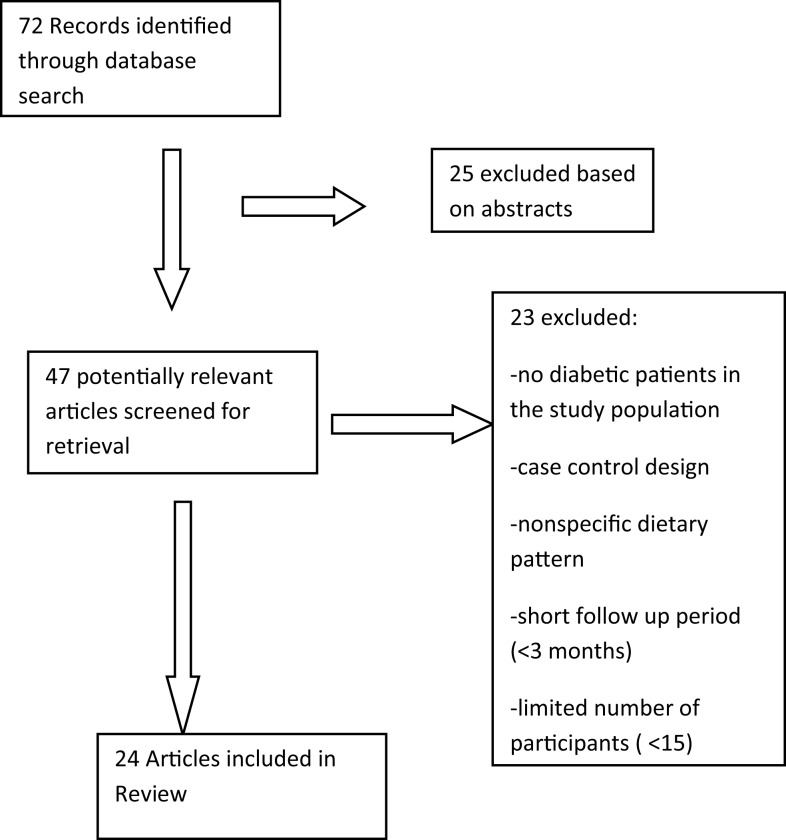
**Flow diagram of the systematic literature search**.

*Exclusion of the abstracts was based on the content and its relevance to our review, and whether it fits our inclusion/exclusion criteria. Most of the abstracts reviewed had full text available*. Of the remaining 47 articles, 23 articles were excluded for either not having diabetic patients in the study population, or for being a case-control design, or having evaluated a non-specific dietary pattern rather than Mediterranean diet, or having short follow up period (<3 months), or a limited number of participants (<15). A total of 24 studies were included by the end of the search (Figure 1).

## Mediterranean Diet and Diabetes Control

Six randomized control studies, and one randomized cross-over study have assessed the effect of Mediterranean diet on parameters of glycemic control including glucose levels, HbA1c, and insulin resistance (Table 1). Out of these seven randomized trials, four were specifically designed to evaluate the effect of Mediterranean diet on glycemic control in T2DM patients (9–12). While in the other three studies, T2DM patients were a subgroup of the design sample (13–15). Most of these studies demonstrated the effectiveness and superiority of Mediterranean diet in glycemic control and insulin sensitivity in T2DM patients when compared to control diets (low-fat diet/usual dietary habits).

**Table 1:** **List of randomized studies on effect of Mediterranean diet on fasting glucose and HbA1c**.

Study author	Study population	Intervention	Follow-up period	End point results
Toobert et al. (9)	279 diabetic postmenopausal women	Control diet (low fat) vs. MLP*	6-month randomized controlled clinical trial	HbA1c: decreased in Mediterranean arm (−0.4% from baseline in MLP group); *P* value <0.01
				No change in control group
Estruch et al. (13)	772 High risk individuals (421 diabetics)	Mediterranean diet (either virgin oil or mixed nuts rich) vs. low-fat diet	3-month randomized controlled clinical trial	Fasting glucose level: decreased in Mediterranean arm (mean difference −7 mg/dl in olive oil Mediterranean diet group compared to low-fat diet group); *P* value <0.017
Shai et al. (14)	322 Moderately obese patients (36 diabetics)	Low-fat restricted calorie diet vs. Mediterranean calorie-restricted diet vs. low carbohydrate non-restricted calorie diet	2-year randomized controlled clinical trial	• **Diabetic patients:**
				**Fasting glucose:** Decreased in Mediterranean arm (by −32.8 mg/dl in the Mediterranean diet compared to baseline and increased by 12.1 mg/dl in low-fat diet; *P* value 0.001; **HbA1c:** No change between groups
				• **Non-diabetic patients**
				**Fasting glucose:** No change between the three groups; **HbA1c:** No change between groups
Esposito et al. (16)	215 patients with newly diagnosed diabetes	Mediterranean diet vs. low-fat diet	4-year randomized controlled clinical trial	Fasting glucose: decreased in Mediterranean arm (−21 mg/dl difference in the Mediterranean diet arm compared to low-fat diet); *P* value <0.01; HbA1c: decreased in Mediterranean arm (−0.6% difference between arms)
Elhayany et al. (11)	259 overweight diabetic patients.	Low carbohydrate Mediterranean diet vs. traditional Mediterranean diet vs. ADA diet	12-month randomized controlled clinical trial	Fasting glucose: no significant change; HbA1c: decreased (difference −0.4% change between low carbohydrate Mediterranean diet and ADA); *P* value 0.002
Itsiopoulous et al. (12)	27 diabetic patients	Mediterranean diet vs. usual diet over 12 weeks then cross over to alternate diet	12 weeks Randomized cross-over interventional study	HbA1c: decreased (−0.3% Change between different groups); *P* value 0.012
Lasa et al. (15)	191 diabetic patients	A low-fat diet (LFD) vs a MD supplemented with 1 L/week of extra-virgin olive oil or a MD supplemented with 30 g/day of mixed tree nuts	12-month randomized controlled clinical trial	HOMA-IR levels: no significant change (all *P* > 0.05)
				Adiponectin/leptin Ratio: increased in all three trial arms (all *P* < 0.05)
				Adiponectin/HOMA-IR ratio: increased in the MD supplemented with olive oil group (*P* = 0.027) and showed a trend toward increase in the MD supplemented with nuts (*P* = 0.069) and the LFD (*P* = 0.061) groups

*Mediterranean Lifestyle Program: comprehensive life style self-management program (including Mediterranean low saturated fat diet, stress management training, exercise and smoking cessation).

### Mediterranean diet effect on plasma glucose and HbA1c

There has been good evidence that adherence to Mediterranean diet might have a beneficial role in glycemic control.

In fact, Esposito et al. conducted a cross-sectional analysis of a population of 901 diabetic outpatients attending diabetes clinic in south Italy. In this study, adherence to Mediterranean diet was inversely associated with both postprandial glucose levels and HbA1c. In multivariate analysis, mean HbA1c and 2-h post-meal glucose concentrations were significantly lower in diabetic patients with high adherence to a Mediterranean-type diet (adherence score 6–9) compared to those with low adherence [difference: HbA1c 0.9%, 95% confidence intervals (CI) 0.5–1.2%, *P* < 0.001; 2-h glucose 2.2 mmol/l, 95% CI 0.8–2.9 mmol/l, *P* < 0.001] (16). Another recent cross-sectional study evaluated 383 participants with type 2 diabetes participating in the PREDIMED trial and looked at the relation between HbA1c levels and adherence to Mediterranean diet. After adjusting for age and sex, there was a trend toward an inverse association between adherence to Mediterranean diet and HbA1c levels, which did not reach statistical significance. Multivariate analysis additionally adjusted for physical activity, smoking, time of evaluation of T2DM, body mass index (BMI), and insulin treatment found similar findings (OR = 0.69, 95% CI 0.17–2.83) (17).

One study looked at the HbA1c in 279 postmenopausal diabetic women who were randomized to receive either usual care (control) or comprehensive life style self-management program that included a Mediterranean diet. There was a significant decrease in the HbA1c in the Mediterranean diet arm (7.43–7.07% *P* = 0.001) as compared to the control (7.4%) after 6 months (9).

Estruch et al. also evaluated the effects of two types of Mediterranean diets (either supplemented with 1 l/week of virgin olive oil or 30 g/day of tree nuts) and a low-fat diet on fasting glucose in 772 high risk patients of which 54.5% are diabetics. After 3 months, both types of Mediterranean diets had significantly lower level of fasting glucose compared to the low-fat diet (13). In another comparative study between three weight loss diets (Mediterranean diet, low-fat diet, and low carbohydrate diet) in 322 moderately obese patients of which 36 are diabetics, Shai et al. found a significant decrease in the fasting plasma glucose level in the diabetic patients receiving the Mediterranean diet (−32.8 mg/dl) compared to low-fat diet. There was no change in the fasting plasma levels in non-diabetic patients receiving any form of the three weight loss diets. Furthermore; there was no difference in the drop in the HbA1c level among patients receiving Mediterranean diet or low-fat diet (−0.5% ± 1.1% vs. −0.4 ± 1.3% *P* = 0.45) (14). The metabolic effects of a Mediterranean diet compared to a low-fat diet were further evaluated by Esposito et al. on 215 patients with newly diagnosed diabetes. After 1 year, both the fasting glucose level and the HbA1c were lower in the Mediterranean diet arm (10).

Another study conducted by Elhayany et al. also evaluated the change in HbA1c and fasting glucose in 259 diabetic patients on stable anti hyperglycemic medication on either low carbohydrates Mediterranean diet, or traditional Mediterranean diet, or the American association diabetes diet (ADA diet). Elhayany et al. found a non-significant decrease in fasting glucose and a significant reduction in HbA1c in patients allocated to the low carbohydrates Mediterranean diet (11).

A recent randomized controlled study studied twenty-seven subjects with type 2 diabetes who were randomly assigned to consume either the Mediterranean intervention diet (*ad libitum*) or their usual diet for 12 weeks, and then cross over to the alternate diet and looked at their HbA1c control. Compared with the usual diet, patients on the *ad libitum* Mediterranean intervention diet had significant drop of their glycosylated hemoglobin from 7.1 to 6.8% (*P* = 0.021) with a significant improvement in the quality of diet (12).

### Mediterranean diet effect on insulin resistance

The association between Mediterranean diet and insulin sensitivity was studied in a Greek adult population. In this study, an inverse association between Mediterranean diet and indices of glucose homeostasis and insulin resistance (assessed by HOMA) was found. However, it is worth noting, that this association was only evident in non-diabetic patients subgroup (18). Another clinical trial evaluated the HOMA-IR in patients with diabetes at 24 months after being randomized either to Mediterranean diet or low-fat diet. In his study, Shai et al. found a greater drop in HOMA index in patients with diabetes adherent to the Mediterranean diet compared to low-fat diet (14). Esposito et al. found a similar inverse relation between Mediterranean diet and HOMA-IR in 215 newly diagnosed type two diabetics after 1 year of either receiving a Mediterranean diet or low-fat diet (10).

In contrast to these findings, there was no difference in HOMA index at 1 year in 116 diabetic patients assigned to either Mediterranean diet or ADA diet on stable antiglycemic medications (11). Lasa et al. had similar results in a recent multicenter parallel trial. This trial was conducted on 191 participants (77 men and 114 women) of the PREDIMED study in order to compare the effects of three different dietary interventions (two Mediterranean diets supplemented with olive oil or mixed nuts and a low-fat diet, LFD) on body weight and glucose metabolism. After 1 year of follow up, increased values of both adiponectin/leptin ratio (*P* = 0.043, *P* = 0.001, *P* < 00.01 for low fat, olive oil, and nut diets respectively) and adiponectin/HOMA-IR ratio (*P* = 0.061, *P* = 0.027, *P* = 0.069 for low fat, olive oil, and nut diets respectively)and significant decreased values of waist circumference were observed in all three diet arms. In both Mediterranean diet groups, but not in the low-fat group these findings were associated with significant reduction in body weight (*P* = 0.347, *P* = 0.03 and *P* = 0.021 for low fat, olive oil and nut diets) (15).

## Diabetes Complication and Risk Factors

In addition to the possible protective effect of Mediterranean diet on glycemic control and homeostasis, growing data suggests potential role for this type of diet in improving cardiovascular risk factors, decreasing mortality, and reducing peripheral artery disease in diabetic patients (10, 11, 13, 19–22).

### Cardiovascular risk factors

The effects of Mediterranean diet on cardiovascular risk factors in diabetic patients was evaluated by Esposito et al. and Elhayany et al. in the above mentioned two controlled studies (10, 11). Both authors were able to show an improvement in systolic blood pressure, HDL level, ratio of total cholesterol to HDL, and triglycerides level in diabetic patients adherent to Mediterranean diet, as well as a significant weight reduction when compared to control diet. Estruch et al. also had similar results concerning cardiovascular risk parameters (systolic blood pressure, cholesterol/HDL ratio) after 3 months of randomizing 772 asymptomatic patients, of which 50% were diabetics, to either low-fat diet (*n* = 257) or Mediterranean diet (rich in virgin olive oil or tree nuts (13). However, this study did not conduct any sub-analysis for the diabetic patients.

Interestingly, Frazer et al. found lowest levels of aminotransferase in the Mediterranean arm in a *post hoc* analysis of a quasi-randomized controlled trial in T2DM patients allocated to Mediterranean diet, ADA diet, or low glycemic diet. This finding made the authors speculate on the potential beneficial effect of the Mediterranean diet on liver steatosis (23). Moreover, the results of 12-week prospective study in 14 obese men with non-alcoholic fatty liver and metabolic syndrome profile showed a great improvement in the steatosis degree (complete regression in 21.4%, and overall improvement in 92.86% of the patients), emphasizing more about the possible beneficial role of Mediterranean diet. It is worth noting that none of the patients included in this study was diabetic (24).

### Cardiovascular disease and total mortality

There are no clinical trials that look solely at the mortality and cardiovascular risk reduction in diabetic patients on Mediterranean diet. However, a number of randomized trials evaluated the effect of the aforementioned diet on the cardiovascular and mortality risk attenuation in the high risk population which included diabetic patients. As a matter of fact, Hodge et al. calculated a personal Mediterranean diet score (MDS) for total of 40,000 participant (of which 2150 had either frank diabetes or high glucose levels) recruited from the Melbourne collaborative cohort study between 1990 and 1994. He collected total and cardiovascular mortality data up to 2003. In his analysis, *Hodge* found an expected increased risk of both total and cardiovascular mortality in diabetic patients (HR 1.4% for men, 1.86%for women). In this study, the hazard ratios (HR) for total mortality per unit of MDS were 0.96 and 0.94%, the HRs for the cardiovascular mortality per unit of MDS were 0.94 and 0.94% in men and women respectively. These data suggest a small reduction in mortality with increased adherence to Mediterranean diet (19).

Singh et al. tested an “Indo Mediterranean diet” in 1000 high risk individuals including 190 diabetic patients. He found a one third reduction in incidence of fatal myocardial infarction and about two third reduction of sudden death from cardiovascular reasons in the Indo Mediterranean diet arm when compared to the controlled diet (20). However, this particular study is not considered to be in support of the beneficial role of Mediterranean diet on mortality and cardiovascular risk especially that an expression of concern has been issued against it (25). The GISSI Prevenzione clinical trial in Italy encouraged 11,323 individuals (1700 diabetics) with history of myocardial infarction to increase their daily consumption of Mediterranean diet components. After comparing the adherence scores, people with highest scores had an odds ratio of mortality of 0.51% when compared to participants with lowest scores emphasizing again on the positive role that Mediterranean diet can exhibit (21).

### Peripheral artery disease

There is not enough data on the effect of Mediterranean diet on peripheral artery disease. However, the association between the risk of peripheral artery disease and adherence to Mediterranean diet was addressed in one cross-sectional study of 944 Italian patients with diabetes. A higher MDS was independently related to a reduction of peripheral artery disease risk, with an odds ratio of 0.44% (95% CI 0.24–0.83) (22).

## Mechanism of Protective Role of Mediterranean Diet in Diabetes

A number of studies were conducted to explore the possible mechanisms of action of a Mediterranean diet in controlling diabetes as well as reducing its cardiovascular risk factors and mortality. There are no adequate studies assessing the mechanism of action of Mediterranean diet in diabetic patients, but some trends can be extrapolated from studies conducted in patients with metabolic syndrome. It seems that each dietary component of the Mediterranean diet have a vital role in the possible protective role.

An anti-inflammatory role has been long thought to be one of the mechanisms through which olive oil rich Mediterranean diet acts. The effects of two different Mediterranean diets on immune cell activation and inflammatory markers in 112 individuals with diabetes (60%) or high cardiovascular risk patients compared to low-fat diet was studied by Mena et al. in a short term interventional trial. After 3 months, expression of adhesion molecules on monocytes and IL6 circulating levels significantly decreased after adherence to both types of Mediterranean diets but not after low-fat diet (26). On the other hand, such an anti-inflammatory effect couldn’t be elicited in a 1-year randomized trial in 101 patients with coronary artery disease including 9% of diabetic patients (27). Another randomized trial conducted on high risk patients including diabetics found a significant drop in CRP levels in patients adherent to Mediterranean diet rich in olive oil as compared to low-fat diet (13). Many studies suggest a role for oleic acid, the predominant monosaturated fatty acid in olive oil, in lowering insulin resistance through increasing adiponectin (28–30), however there are contradictory results concerning this particular effect on insulin resistance (31, 32). The antioxidant characteristic of its rich content of fruits, vegetables and cereals seems to play a major role in its protective potential. Interestingly, Evan et al. demonstrated how prolonged oxidative stress was associated with increased beta cell dysfunction as well as insulin resistance (33). Furthermore, two clinical trials showed that administration of antioxidant vitamins improved insulin sensitivity (34, 35). Sanchez-Moreno et al. reported a reduced amount of F2 isoprostane, a marker of oxidative stress and increased vitamin C levels in healthy individuals on a typical Mediterranean plate (36). Recently, Barona et al. was able to show that Mediterranean-style low-glycemic-load diet for 12 weeks in thirty five women with metabolic syndrome and high LDL levels (>100 mg/dl) significantly reduced oxidized LDL (12% in Mediterranean diet) (37). Moderate alcohol consumption, as part of the dietary pattern in the Mediterranean diet seemed to have a beneficial effect on insulin resistance by increasing adiponectin levels (38) as it was observed on multiple epidemiological studies (39–41). This was supported by a cross-sectional study conducted on 987 diabetic women, a subsample of the Nurse Health Study that demonstrated that patients with highest adherence to Mediterranean diet had 23% higher levels of adiponectin compared to those with lowest Mediterranean diet adherence (42).

Another important component of the Mediterranean diet is dietary fiber which is believed to induce satiety and thus reduce caloric intake (43–45).

## Conclusion

In this systemic review, the potential positive role and health benefits of Mediterranean diet on diabetes control and its cardiovascular complications were explored. Despite the difficulty to assess adherence to such a diet, the limited number of studies focusing on the full effects of Mediterranean diet in diabetic subjects, the strict exclusion/inclusion criteria followed, there is good evidence that adherence to Mediterranean diet seems to have a protective role on glycemic control as reflected by reduced HbA1c and lower fasting levels in addition to decreased insulin resistance and mortality. Decreasing oxidative stress, inflammation, and insulin resistance are all possible mechanism by which Mediterranean diet pauses as a protective dietary pattern. Concerning cardiovascular risk modification, there is some cardiovascular benefit of adhering to a Mediterranean diet in diabetic patients; although there are no studies looking at the cardiovascular outcome in diabetic patients only. This fact necessitates further studies to assess the effect of adherence to Mediterranean diet on cardiovascular risk in diabetic patients.

## Conflict of Interest Statement

The authors declare that the research was conducted in the absence of any commercial or financial relationships that could be construed as a potential conflict of interest.

## Appendix

Keyterms used in database search:

Mediterranean Diet
Dietary Pattern
Type II Diabetes
Glycemic Control
HbA1c
Insulin Resistance
Insulin sensitivity
HOMA
Cardiovascular Disease
Coronary Artery Disease
Peripheral Arterial Disease
Mechanism of Action
Antioxidant.
